# Supplementary material for: Trends of Antimicrobial Susceptibility in Clinically Significant Coagulase-Negative Staphylococci Isolated from Cerebrospinal Fluid Cultures in Neurosurgical Adults: a Nine-Year Analysis
Source: Microbiol Spectr. 2022 Feb 9;10(1):e01462-21. doi: 10.1128/spectrum.01462-21 (PMC8826829; doi:10.1128/spectrum.01462-21)
Supplement: SUPPLEMENTAL FILE 1 — Supplemental material. Download SPECTRUM01462-21_Supp_1_seq10.pdf, PDF file, 0.1 MB [file spectrum01462-21_supp_1_seq10.pdf]

Diagnostic criteria of contaminated episodes

Contaminated episodes were defined as not infected episodes and met one of the following criteria. I. The patient had no clinical features (1, 2). II. The patient had clinical features, but the bacteria were not pathogens (3); there were three subsets of this category: IIa. the improvement occurred without susceptible antimicrobial agents (4); IIb. the duration of the susceptible antimicrobial agent was less than 72 hours for medical reasons, and no reinfection occurred (5); and IIc. the susceptible antimicrobial agent was ineffective in the patient without special situations, including poor wound healing, implants, cerebrospinal fluid leak, abscess, and ventriculitis, and the course was self-limiting without adjusting the antimicrobial agent (6). III. The patient had clinical features, and bacteria other than the isolated bacteria were the pathogen because of the high contamination rate in cerebrospinal fluid cultures and low etiologic diagnosis rate in healthcare-associated ventriculitis and meningitis (7). In this situation, the susceptible antimicrobial agent was ineffective in the patient without special situations, and the improvement occurred after adjusting the antimicrobial agent.

References

1. Boysen MM, Henderson JL, Rudkin SE, et al. Positive cerebrospinal fluid cultures after normal cell counts are contaminants. J Emerg Med. 37(3). United States,2009. 251-6.
2. Steinbok P, Cochrane DD, Kestle JR. The significance of bacteriologically positive ventriculoperitoneal shunt components in the absence of other signs of

shunt infection. J Neurosurg. 84(4). United States,1996. 617-23.

3. Mount HR, Boyle SD. Aseptic and Bacterial Meningitis: Evaluation, Treatment, and Prevention. Am Fam Physician. 96(5). United States,2017. 314-322.
4. Forgacs P, Geyer CA, Freidberg SR. Characterization of chemical meningitis after neurological surgery. Clin Infect Dis. 32(2). United States,2001. 179-85.
5. Zarrouk V, Vassor I, Bert F, et al. Evaluation of the management of postoperative aseptic meningitis. Clin Infect Dis. 44(12). United States,2007. 1555-9.
6. Bihan K, Weiss N, Théophile H, et al. Drug-induced aseptic meningitis: 329 cases from the French pharmacovigilance database analysis. Br J Clin Pharmacol. 85(11) ,2019. 2540-2546.
7. Srihawan C, Castelblanco RL, Salazar L, et al. Clinical Characteristics and Predictors of Adverse Outcome in Adult and Pediatric Patients With Healthcare-Associated Ventriculitis and Meningitis. Open Forum Infect Dis. 3(2) ,2016. ofw077.
